# Nitric oxide contributes to high-salt perception in a blood-sucking insect model

**DOI:** 10.1038/s41598-017-15861-0

**Published:** 2017-11-14

**Authors:** Agustina Cano, Gina Pontes, Valeria Sfara, Diego Anfossi, Romina B. Barrozo

**Affiliations:** 1 Grupo de Neuroetología de Insectos Vectores, Laboratorio Fisiología de Insectos, Facultad Ciencias Exactas y Naturales, Departamento Biodiversidad y Biología Experimental, Universidad de Buenos Aires; Instituto Biodiversidad Biología Experimental y Aplicada (IBBEA), CONICET - UBA, Ciudad Universitaria, Pabellón 2, CP1428 Buenos Aires, Argentina; 2 Instituto de Investigación e Ingeniería Ambiental, Universidad Nacional de San Martín (3iA – UNSAM), Av. 25 de mayo y Francia, CP1650 San Martín, Buenos Aires Argentina

## Abstract

In all organisms, salts produce either appetitive or aversive responses depending on the concentration. While low-salt concentration in food elicits positive responses to ingest, high-salt triggers aversion. Still the mechanisms involved in this dual behavior have just started to be uncovered in some organisms. In *Rhodnius prolixus*, using pharmacological and behavioral assays, we demonstrated that upon high-salt detection in food a nitric oxide (NO) dependent cascade is activated. This activation involves a soluble guanylate cyclase (sGC) and the production of cyclic guanosine monophosphate (cGMP). Thus, appetitive responses to low-salt diets turn to aversion whenever this cascade is activated. Conversely, insects feed over aversive high-salt solutions when it is blocked by reducing NO levels or by affecting the sGC activity. The activation of NO/sGC/cGMP cascade commands the avoidance feeding behavior in *R. prolixus*. Investigations in other insect species should examine the possibility that high-salt aversion is mediated by NO/sSG/cGMP signaling.

## Introduction

The taste sense helps animals to evaluate the quality of food, favoring the ingestion of nutrients and avoiding the consumption of harmful or toxic compounds. Sugars, amino acids and low-salt concentrations indicate the presence of nutritious foods and stimulate feeding, whereas bitter compounds and high-salt concentrations are aversive molecules that prevent ingestion^1^.

Interestingly, salt perception can elicit opposite feeding behaviors according to its concentration. As expressed above, the detection of low-salt concentrations in food generally trigger feeding acceptance while high-salt concentrations are generally rejected^2–4^. Because salts are essential nutrients, their detection is crucial. Salts such as sodium chloride participate in vital physiological functions maintaining the internal homeostasis and neuronal transmission^5,6^. Feeding on deficient or excessive salt sources could drive animals to physiological disorders. Taste is then tuned to accept adequate concentrations of salts, according to each animal’s necessity. In this way, “tasteful” usually matches with “low” salt concentrations in food.

Blood-sucking insects feed on vertebrates’ blood, where salts are major components. Both, salt identity and concentration are key factors regulating feeding decisions of blood-sucking insects^7–10^. To examine the fundamental question on how low-salt and high-salt perception are discriminated in insects, we chose the kissing bug *Rhodnius prolixus*. Besides serving as vectors of Chagas disease, they have proven to be excellent models for physiology studies since the early 30s^11,12^. The taste system provides kissing bugs with adequate information about the amount of salt in the food. *R. prolixus* avoids feeding on solutions below or above an optimal range of salt concentration (0.1–0.15 M)^9^. Biting behavior and the pumping activity of the cibarial pump (a large structure associated to a complex of muscles located in the head of the insect) are finely controlled by salt concentration^9^. *R. prolixus* perceive low-salt, no-salt (distilled water) and high-salt solutions as different gustatory information, as revealed the electromyogram recordings (EGMs) of the cibarial pump. High- and no-salt solutions are rejected by insects; however, both saltiness conditions evoke different feeding behaviors. Moreover, sudden changes in the salt concentration of the diet during feeding interfered with the ingestion, evincing that these bugs perform a continuous gustatory monitoring of the incoming food during feeding^9^.

The mechanism controlling the concentration-dependent behaviors elicited by salts is still an open question that researchers have begun to understand in some animals^2–4,13,14^. In both, mammals and insects, two separate taste pathways have been proposed for salt sensing: an appetitive (tuned to low salt detection) vs. an aversive pathway (for high salt detection). Detection of low-salt concentrations in mammals is mediated by taste receptor cells expressing ENaC receptors^2^, and in insects by ppk11, ppk19 and IR76a receptors^3,4^. High-salt sensing in mammals seems not involve specifically dedicated taste receptor cells but instead through the recruitment of two populations of cells tuned to detect bitter and sour^15^. Conversely, in fruit flies, specific taste neurons are sensitive to detect high-salt concentrations, though they also detect bitter compounds^13^. Two genes, *ppk19* and *sano*, expressed in high-salt sensing neurons of fruit flies larvae are required for high-salt detection^16^. Still, the mechanisms and signaling pathways involved in high-salt detection of animals in general are barely understood.

In this work, we asked whether nitric oxide (NO) signaling is involved in the detection of high-salt levels in food. NO is a free gas signaling molecule that activates a soluble guanylate cyclase (sGC) that produces cyclic guanosine monophosphate (cGMP) as a second messenger^17^. NO is generated by a Ca^2+^-dependent NO synthase (NOS) which using NADPH as co-factor converts L-arginine into NO and citrulline^18^. Calcium influx through voltage-gated ion channels, together with calcium release from intracellular stores are thought to trigger NO synthase activation^19^. The biological role of NO in the peripheral and central nervous system has been extensively studied in mammals and insects^17,19^. Particularly, NO participates in several sensory processes including gustation, thus, it can be found in taste receptor cells of mammals^20,21^ and of insects^22,23^. In *R. prolixus*, NO was shown to be involved in chemosensory processes^24,25^. Topical application of a NO donor (S-nitroso-N-acetylcysteine, SNAC) over kissing bugs’ antennae significantly decreased the feeding response over a living host^24^.

Knowing that taste detection of low-salt concentration in artificial diets triggers feeding in *R. prolixus*, we propose that whenever salt concentration rises beyond a threshold of tolerance, a NO/sGC/cGMP pathway is activated in taste receptor cells. Thus, the activation of this signaling cascade might prevent *R. prolixus* from ingesting solutions with high-salt content. In order to test this hypothesis we examined the effect of a NO donor, a NO scavenger, a sGC inhibitor and a cGMP analogue on the feeding behavior of kissing bugs while feeding on low- and high- salt solutions.

## Results

We analyzed the role of the NO/sGC/cGMP cascade in salt perception in kissing bugs. First, we investigated the effect of the administration of a NO donor (SNAC), a sGC inhibitor (1H-[1,2,4]oxadiazolo[4,3-a]quinoxalin-1-one, ODQ) or a cGMP analogue (8-bromoguanosine 3′,5′-cyclic monophosphate, 8-Br-cGMP) on the feeding behavior of insects offered with a low-NaCl feeding solution (low-Na). Second, we analyzed if the antifeedant response to a high-NaCl solution (high-Na) can be reverted after interfering with the NO/sGC/cGMP cascade by using a NO scavenger (2-phenyl-4,4,5,5-tetramethylimidazoline-1-oxyl 3-oxide, PTIO) or ODQ.

### Feeding on low-Na diet: NO/sGC/cGMP activation produces a false high-salt perception

**Figure 1:**
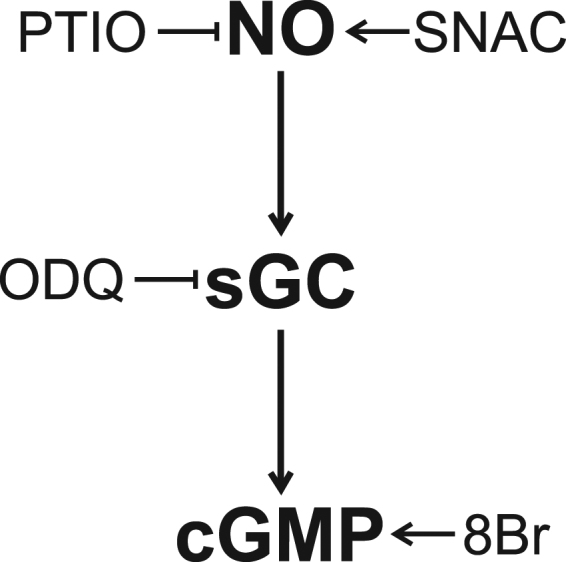
Drugs’ effects over the NO/sGC/cGMP cascade. Nitric oxide (NO) activates the guanylate cyclase (sGC) that produces cyclic guanosine monophosphate (cGMP). SNAC is a NO donor, ODQ inhibits the sGC activity, 8-Br-cGMP (8Br) is a cGMP analogue, PTIO is a NO scavenger.

**Figure 2:**
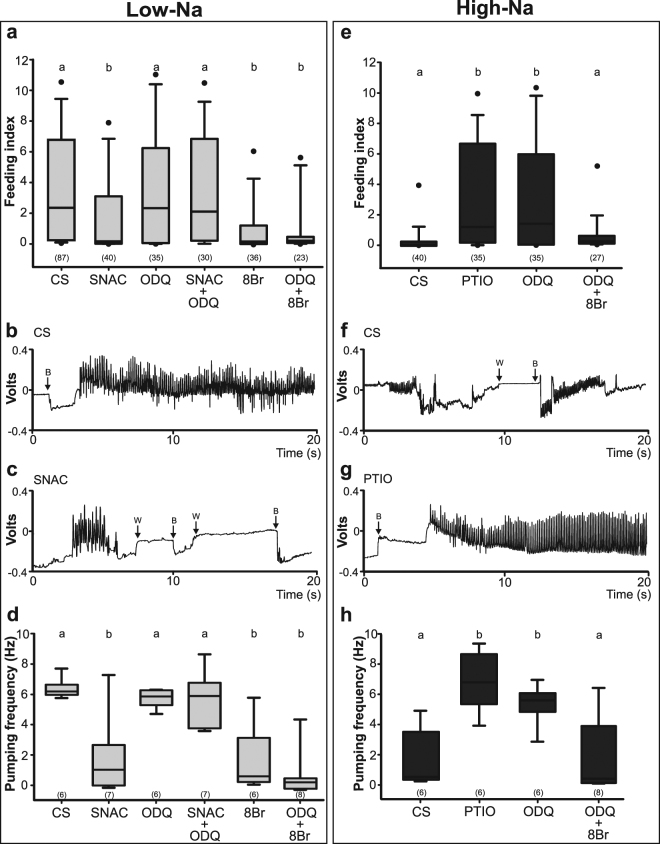
Low-salt versus high-salt perception and the role of the NO/sGC/cGMP cascade. Low-salt solution (**a–d**) or high-salt solution (**e–h**) were offered to insects. (**a,e**) Feeding index of insects previously treated with control solution (CS), SNAC (NO donor), ODQ (sGC inhibitor), 8-Br-cGMP (cGMP analogue) or PTIO (NO scavenger). (**b,c,f,g**) EMG examples of insects feeding on low-Na when previously treated with CS (**b**) or SNAC (**c**) and of insects feeding on high-salt but previously treated with CS (**f**) or PTIO (**g**). EMG recordings lasted 10 minutes, although only 20 seconds are shown. (**d,h**) Pumping frequency of the cibarial musculature (in Hz, pumps per second) of treated insects with CS, ODQ, SNAC, 8-Br-cGMP or PTIO. Different letters denote significant differences (p < 0.05). Numbers of insects used are shown in brackets. B = insertion of mouthparts in the feeder, W = withdrawal of mouthparts.

SNAC and 8-Br-cGMP can activate the NO/sGC/cGMP pathway at different levels (see Fig. 1). Control insects (CS, represents the average of all control groups pooled due to the absence of differences among them, H_2_ = 1.7, p = 0.4271) fed as much as 2 times their own weight (Fig. 2a). However, insects treated with SNAC or 8-Br-cGMP fed significantly less than controls when a low-Na solution was offered as food (Fig. 2a, H_5_ = 28.79, p < 0.0001; post hoc comparisons, CS vs. SNAC, 8-Br-cGMP p < 0.0001). Moreover, the simultaneous treatment with SNAC and ODQ reverted this inhibition, evincing that sGC participates in the signaling pathway (Fig. 2a, post hoc comparison, SNAC vs. SNAC + ODQ, p = 0.01). Feeding was not affected when ODQ was administrated alone and insects fed similarly to controls (Fig. 2a, post hoc comparison, p = 0.94). Moreover, the sGC involvement was additionally supported when insects were treated with ODQ + 8-Br-cGMP. Following this treatment, insects significantly reduced feeding with respect to controls or ODQ treated insects (Fig. 2a, post hoc comparison, ODQ + 8-Br-cGMP vs. CS, ODQ, p < 0.01). Note additionally that ODQ + 8-Br-cGMP and 8-Br-cGMP treated insects were not statistically different (Fig. 2a, post hoc comparison, ODQ + 8-Br-cGMP vs 8-Br-cGMP, p = 0.6).

In parallel, the activity of the cibarial pump of insects feeding on the appetitive low-Na solution was registered by means of EMGs. Figure 2b and f depict examples of EMG recordings of insects offered with the low-Na solution and a high-Na solution respectively. The visual inspection of EGMs reveals clear differences in the pumping activity at both Na conditions. At low-Na, the pumping pattern was regular exhibiting one or two piercing events, while at high-Na it became irregular and involved numerous piercing events.

The pumping frequencies of insects from the different treatments are summarized in Fig. 2d. Concordantly with the results shown in Fig. 2a, SNAC or 8-Br-cGMP treated insects showed pumping frequencies significantly reduced (Fig. 2d, H_5_ = 24.01, p = 0.0002; post hoc comparisons CS vs. SNAC, 8-Br-cGMP, p < 0.01). As an example, the EMG recording of an individual treated with SNAC is shown in Fig. 2c. Notably the pumping pattern over low-Na of this group is comparable to individuals feeding on high-Na (compare EGMs of Fig. 2c and f). The antifeedant effect was again reverted by the addition of ODQ to the SNAC treatment (Fig. 2d, post hoc comparison, SNAC vs. SNAC + ODQ, p = 0.048), evincing that the activation of the NO/sGC/cGMP pathway participates in the feeding response. This result was further confirmed by treating insects with ODQ + 8-Br-cGMP, here, insects exhibited significantly low pumping frequencies with respect to CS or ODQ treatments (Fig. 2d, post hoc comparison, ODQ + 8-Br-cGMP vs. CS, ODQ, p < 0.001), but exhibited no differences with 8-Br-cGMP treated animals (Fig. 2d, post hoc comparison, p = 0.29). Thus, NO/sGC/cGMP activation can trigger a false aversive behavior in response to an appetitive stimulus, producing a reduction in the ingested volume through a decrease in the pumping activity.

### Feeding on high-Na diets: NO/sGC/cGMP inhibition produces a false low-salt perception

PTIO and ODQ can inhibit the NO/sGC/cGMP pathway at two different levels (see Fig. 1). Control insects (CS) avoided feeding on high-Na, this group showed significantly lower ingested volumes than insects offered with low-Na (Fig. 2a and e, CS low-Na vs. CS high-Na, W = 1510, p < 0.0001). However, when treated with either PTIO or ODQ, insects ignored the presence of high salt and fed as much as they did on the low-Na solution (Fig. 2e, H_3_ = 29.99, p < 0.0001, post hoc comparisons CS vs. ODQ, PTIO, p < 0.000003). Consistently, EMG recordings showed that the low pumping frequency of insects feeding on the high-Na solution (Fig. 2f) increased from around 1 Hz to 6 Hz when treated with PTIO (Fig. 2g,h) and to 5.5 Hz when insects were treated with ODQ (Fig. 2h, H_3_ = 14.72, p = 0.002, post hoc comparisons CS vs. ODQ, PTIO, p < 0.04). Lastly, the ODQ effect was reverted when insects were treated simultaneously with ODQ and 8-Br-cGMP (Fig. 2e,h, post hoc comparisons, ODQ vs ODQ + 8-Br-cGMP, p = 0.02 and p = 0.03, respectively). ODQ + 8-Br-cGMP treated insects showed similar feeding behaviors to CS insects (Fig. 2e,h, post hoc comparisons, CS vs. ODQ + 8-Br-cGMP, p = 0.06 and p = 0.98, respectively). Moreover, we compared the feeding values (i.e. feeding index or pumping frequency) of PTIO and ODQ treated insects feeding on high-Na against those of CS insects offered with low-Na solution. These comparisons revealed no significant differences (H_2_ = 0.63, p = 0.7298 and H_2_ = 4.54, p = 0.1034, for the feeding index and pumping frequency respectively), suggesting that NO/sGC/cGMP cascade is the sole active aversive pathway.

Altogether these results show that the artificial inactivation of the NO/sGC/cGMP cascade in the taste system of insects converted an aversive feeding response to high-Na into an appetitive response. Conversely, the artificial activation of this cascade turns an appetitive response to low-Na into an aversive response.

### The NO/sGC/cGMP pathway is specific for salts

**Figure 3:**
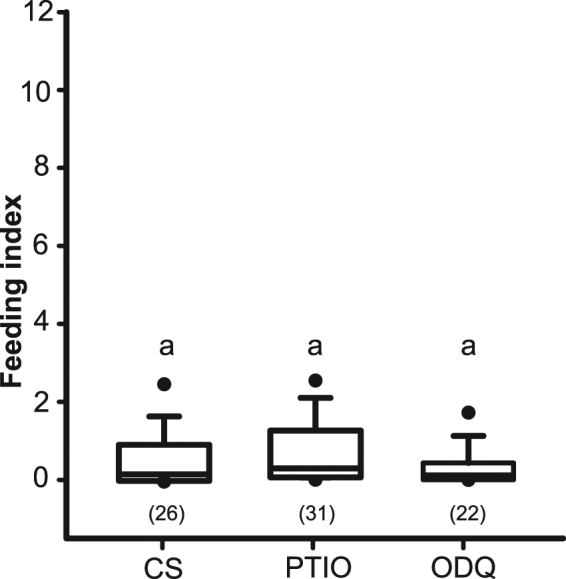
Caffeine aversion and the role of NO/sGC/cGMP cascade. Feeding index of insects offered with a caffeine solution in the artificial feeder after CS, PTIO and ODQ treatments. Similar letters denote no statistical differences (p > 0.05). Numbers of insects used are shown in brackets.

Kissing bugs do not feed on a low-Na solution if the bitter alkaloid caffeine is also present^26^. We tested here if this bitterness mediating feeding inhibition is also controlled by the NO/sGC/cGMP pathway (Fig. 3). PTIO and ODQ treatments did not modify the deterrent effect to caffeine (H_2_ = 4.3, p = 0.1162). This result strongly suggests that the feeding inhibition caused by caffeine must occur via a different and independent pathway (not studied in this work).

## Discussion

In this work, we demonstrated that the activation of the NO/sGC/cGMP pathway is implicated in avoidance of high-Na concentrations in *R. prolixus*. By pharmacologically manipulating the NO signaling cascade, while insects fed over low-Na or high-Na solutions, we uncovered its function. On the one hand, NO increment (by means of the NO donor SNAC) decreased feeding of *R. prolixus* over an appetitive low-Na solution. Moreover, this effect was suppressed when simultaneously treating insects with the sGC inhibitor (ODQ), showing that sGC is the molecular target of NO. A significant feeding reduction over the low-Na was also produced by the 8-Br-cGMP treatment alone or together with ODQ, thus, cGMP production seems to be the consequence of the NO/sGC cascade activation. On the other hand, insects accepted feeding on high-Na whenever NO levels and the sGC activity were reduced. Moreover, under these conditions, insects fed as much as did control insects at low-Na. Consequently, the activation of NO/sGC/cGMP signaling cascade upon high-Na taste stimulation could be responsible to initiate the antifeedant response. In contrast, the NO/sGC/cGMP cascade seems not to be the mechanism involved in the detection of other aversive molecules such as the caffeine. As we have shown, treatments with PTIO and ODQ did not modify the aversive behavior towards caffeine. It is not possible to discard the existence of another intracellular sGC -not inhibited by the ODQ- involved in the aversive response to caffeine. Vermehren-Schmaedick and colleagues^27^ showed that atypical sGC are required to process taste responses in *Drosophila melanogaster*. Two atypical sGC are involved in bitter avoidance behavior or in sucrose attraction response in larvae and adults. Although these atypical sGC are activated by O_2_ instead of NO^27^.

NO is a signaling molecule that plays essential roles in sensory systems -including olfaction, vision, mechanosensation and taste- as well as in other processes^17,19,28–30^. NO has been linked to food intake in mammals, where NO exerts its modulatory role by attenuating food ingestion^31,32^ and the same was suggested for insects^23^. In locusts, by modulating the responsiveness of salt receptors cells (i.e. decreasing the firing rate), NO could change food acceptance for NaCl, making food rich in NaCl acceptable, which would otherwise be rejected^23^. NO also modulates the activity of appetitive signaling cells like the sugar receptor cells in blowflies^33^ and locusts^23^. If NO affects the activity of peripheral taste cells, it might be produced close to its targets. NO production has been found in the taste receptor cells of vertebrates and insects^20–22,34^ and also in the vicinity of taste cells of insects^23^. NO modulation can involve cGMP-independent pathway^23,35^ or a cGMP-dependent pathway^33,35,36^. Although there is sparse evidence showing the role of cGMP in taste signaling in insects and mammals^36,37^, it was suggested to be the second messenger for the sugar sensory cells of blowflies^30,38^ and locusts^35^. cGMP was also related to signal transduction of sugar and bitter tastes in mice^39,40^. cGMP acting as a second messenger may directly gate ion channels or facilitate protein phosphorylation^17^. Future investigations will have to localize the presence of NOS and sGC in the gustatory neurons of *R. prolixus*, as expected from findings in chemosensory cells of vertebrates^20,21,41^, and invertebrates^27,42–44^. Furthermore, to determine cGMP levels by means of *in vivo* measurements^43^ upon high-Na stimulation will provide more evidence about its role as messenger.

Salts are main constituents of the vertebrate blood, and its concentration regulates feeding decisions of kissing bugs, and of other blood feeders^7–10^. The optimal appetitive salt (NaCl or KCl) concentration for *R. prolixus* is around 0.1–0.15 M as long as ATP is present^9^. Not surprisingly, this salt concentration has an osmolarity equivalent to that of the vertebrate plasma. However, it is salt concentration, but not osmolarity which appears to be a key factor to trigger feeding in *R. prolixus* ^9^. In contrast, salt content in the food that exceeds the tolerance limits of kissing bugs has an antifeedant effect (this work: 0.2 M; ref.^9^: 0.3 M). *R. prolixus* also avoid feeding on no-salt content solutions (even if ATP is present), however, biting and the pumping performance of insects to no-salt conditions is markedly different from high-Na diets^9^.

**Figure 4:**
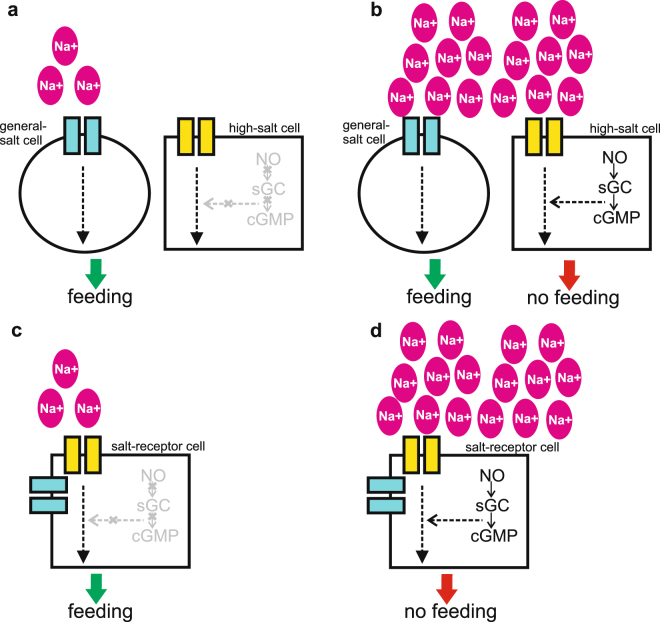
Proposed models for salt perception. Two models are proposed for sensing low and high concentration of salts in food. (**a,b**) In the 2 cells model, one general-salt cell is tuned to a broad spectrum of salt concentrations and the other only responds to high salt levels, the high-salt cell. At low salt levels (**a**), the general-salt cell triggers a feeding message. At high-salt levels (**b**), the NO/sGC/cGMP cascade is activated in the high-salt cell resulting in a “no feeding” message to the brain, which overwhelms the “feeding” message produced by the general-salt cell. (**c,d**) In the 1 cell model, only one salt-receptor cell is enough for salt concentration discrimination. At low salt, the NO/sGC/cGMP cascade is inactive, and this salt detector produces a “feeding” signal upon low salt levels. At high salt, the NO/sGC/cGMP cascade is activated within this cell and the feeding message turns to “no feeding”. Putative sodium receptors are drawn as square boxes.

Salt detection in *R. prolixus* seems to occur through 8 uniporous sensilla located in the anterior region of the pharynx^9,26^. Thus, when the insect takes a sip of the feeding solution the pharyngeal sensilla become in contact with the incoming food. When salt content is appropriate (i.e. low salt) the gustatory neurons housed in these sensilla may inform the brain. Consequently the cibarial musculature starts pumping to fill the gut (this work and ref.^9^). Therefore, gustatory information collected by pharyngeal sensilla could supply the brain with the necessary information for controlling the sucking activity of insects. At least two receptors are related to salt sensing in *R. prolixus*, one sensitive to amiloride blockade and the other insensitive^9^. In the fruit fly *D. melanogaster*, two cells are responsible for low- and high-salt detection. Both cells transmit direct information to the brain about the amount of salts in food, i.e. one cell is tuned to low-salt concentrations and the other to high salt^3,13^. Considering fruit flies findings and our results, we propose two hypothetical models for salt concentration discrimination in *R. prolixus* (Fig. 4). In the first model (Fig. 4a,b), two salt detectors are involved: a broad-spectrum salt cell tuned to a wide range of salt concentration (general-salt cell) and a high-salt cell tuned only to high-salt concentrations. Upon low-salt detection the general-salt cell triggers a “feeding” message (Fig. 4a). At high-salt concentrations, the activation of the high-salt cell together with the NO-dependent cascade would overwhelm the general-salt cell response, resulting in salt rejection or “no feeding” message (Fig. 4b). Remarkably whenever the NO/sGC/cGMP cascade is artificially blocked, feeding over high-salt solutions could only occur if the general-salt cell produces a “feeding” message independently of the salt concentration and NO levels. In the second model (Fig. 4c,d), a single neuron is enough for salt concentration discrimination. Whenever salt concentration in food is adequate (low) a “feeding” signal is produced (Fig. 4c) while the NO/sGC/cGMP cascade is inactive within these salt cells (Fig. 4c). But when salt levels in food are high enough (Fig. 4d), the NO/sGC/cGMP cascade becomes active modulating the activity of these receptor cells (likewise to locusts, see above^23^). NO synthesis could be the consequence of an increase of intracellular Ca++, thus, Ca++ bound to a calmodulin can activate the nitric oxide synthase (NOS) to produces NO^17^. As we showed, the mere inactivation of the NO/sGC/cGMP cascade, by reducing NO levels or by affecting the sGC activity, can lead *R. prolixus* to feed over high-salt solutions which otherwise would be avoided. Conversely, a feeding response initiated by low salt levels can turn into a “no feeding” behavior whenever the NO/sGC/cGMP cascade is activated. NO, acting as neurotransmitter or neuromodulator, can modulate the firing rate of taste cells, i.e. increasing or decreasing, and thus changing the peripheral message^23,33^ delivered to central areas in the brain.

Although belonging to different taste modalities, sweet and bitter compounds also drive opposite behaviors. Recent studies have started uncovering the mechanisms behind these dual behaviors to sugar and bitter molecules in fruit flies^45^. Bitter compounds not only cause aversive responses via the activation of specific taste neurons but also through the inhibition of sugar receptor cells. This inhibition can occur at the periphery by direct interference with sugar reception or transduction mechanisms^46–48^. Besides, in the presence of bitter compounds GABAergic neurons through presynaptic control can suppress the output of sugar receptor cells^49^.

Whether a dual taste pathway (2 cells model, Fig. 4a,b) or a single pathway (1 cell model, Fig. 4c,d) are involved in salt concentration perception in *R. prolixus* needs further investigation. Despite this, we demonstrated an unprecedented case for the role of NO/sGC/cGMP cascade whose activation can turn an appetitive feeding response into an aversive response and vice versa.

## Materials and Methods

### Insects

Fifth-instar larvae of *R. prolixus* were used throughout the experiments. Animals were reared in the laboratory, at 28 °C, ambient relative humidity and under a 12 h: 12 h light: dark cycle. Following ecdysis as fifth-instars, insects were kept unfed for 15 ± 2 days and then used in the experiments.

All experiments were carried out between the 1^st^ and 6^th^ hour of the scotophase^50–52^.

### Treatments

Before analyzing the feeding performance of *R. prolixus* offered with different salt solutions, insects were treated with a NO donor (SNAC), an inhibitor of the soluble guanylate cyclase (ODQ), a cell permeable cGMP analogue (8-Br-cGMP) or a NO scavenger (PTIO) (Fig. 1), and their respective controls. The efficiency of all these drugs was probed in different arthropods^24,25,36,53–57^. Treatments were administrated by ingestion (see below).

SNAC (S-nitroso-N-acetylcysteine) was prepared as the acid-catalyzed nitrosation of N-acetylcysteine by using NaNO_2_ in 0.1% EDTA, at pH = 2, as previously described in^24^. Next, the solution was neutralized with 0.5 M NaOH to achieve a pH = 7 and further diluted in 0.15 M NaCl to obtain a final concentration of 0.2 M SNAC. ODQ (1H-[1,2,4]oxadiazolo[4,3-a]quinoxalin-1-one) was dissolved in 0.5% of dimethyl sulfoxide (DMSO) and in 0.15 M NaCl, obtaining a final concentration of 1 mM. 8-Br-cGMP (8-bromoguanosine 3′,5′-cyclic monophosphate) was prepared at 2 mM in 0.15 M NaCl. PTIO (2-phenyl-4,4,5,5-tetramethylimidazoline-1-oxyl 3-oxide) was dissolved in 0.5% DMSO and diluted in 0.15 M NaCl, obtaining a final concentration of 1 mM.

The control solution for the SNAC treatment contained 0.15 M NaCl, N-acetylcysteine and 0.1% EDTA. As control solutions for 8-Br-cGMP, ODQ and PTIO treatments, a 0.15 M NaCl solution with or without 0.5% DMSO were used accordingly.

All chemicals were purchased from Sigma-Aldrich (St Louis, MO, USA). Solutions were prepared daily.

### Artificial feeder: the feeding index

The feeding behavior of kissing bugs was examined by measuring the ingested volume of insects over different feeding solutions. The artificial feeder described previously by Pontes *et al*.^26^ was used. Briefly, it consisted of a cylindrical feeding recipient (2 ml) with its lower opening closed with a biting membrane of latex, which was filled with different feeding solutions. Solutions were heated to 35 ± 1 °C, thus, the biting membrane match the average temperature of a vertebrate host skin. Separately, a plastic vial (10 ml) whose upper opening was covered with a tissue mesh contained one insect. A piece of filter paper inside this recipient helped insects to climb in order to reach the tissue mesh. Insects could easily perforate the tissue mesh and the biting membrane with their mouthparts to have access to the feeding solution. For treatment administration, each insect was allowed to feed on the corresponding treatment solution for 1 minute. If the animal discontinued ingestion before this time, it was discarded. Shortly after (0.5–1 minute), the insect was transferred to a contiguous feeder containing the feeding solution (see below). The experiment started once the insect’s recipient and the feeder were put in contact and lasted for 10 minutes.

Each insect was weighed before the treatments (Wi), after treatment administration (Wt) and at the end of the feeding assay (Wf). A feeding index (FI) was calculated as the mass gained at the end of assay, Wf - Wt, and normalized to the Wi to avoid biases due to mass differences among insects as follows:

$$Feeding\,index\,(FI)=\frac{{W}_{f}-{W}_{t}}{{W}_{i}}$$

### Artificial feeder: cibarial pump electromyogram recordings

Besides the mass gained by the insects, the activity of cibarial pump muscles of insects feeding on the different solutions in the artificial feeder (detailed above) was registered by means of electromyogram recordings (EMGs). The methodology used is detailed in^9,58^. Briefly, one silver electrode was fixed to a conductive metallic mesh where the insect was posed during feeding. A second silver electrode was placed inside the feeding recipient of the artificial feeder in contact with the solution. Both electrodes were connected to a differential amplifier (Dagan Corporation, EX-1). When the insect inserted the mouthparts in the feeding recipient, the circuit closed generating a base conductance. Once the animal began pumping, the baseline signal changed as a result of the contractions produced by the cibarial muscles. Because insects remained completely immobile while feeding, exclusively the contractions of the cibarial pump musculature were registered. Recorded signals were filtered by means of low pass filter (30 Hz), amplified (x 200) and digitalized with aid of the A/D converter of the oscilloscope (Tektronix TDS 210) connected to a PC. Data were analyzed by means of software designed ad hoc.

The pumping frequency was calculated as the number of peaks (i.e. each peak represented a muscle contraction^58,59^) during the biting time (i.e. the effective time that the animal kept the mouthparts inside the feeder) (for further details see^9^).

### Feeding solutions

The feeding performance of insects was evaluated using three feeding solutions. All solutions contained the phagostimulant ATP (0.001 M) to ensure the motivation of insects to feed. The following combinations were prepared: 1- low-NaCl (0.15 M); 2- high-NaCl (0.2 M); 3- caffeine (0.005 M caffeine in 0.15 M NaCl).

ATP was purchased from Sigma-Aldrich (St Louis, MO, USA), NaCl and caffeine from Biopack (Buenos Aires, Argentina).

### Data analysis

Data were analyzed by means of non-parametric statistics and represented as box-plots (i.e., the graphs included the median and the 95th percentiles). Statistical differences among treatments were assessed by using Wilcoxon for paired samples and Kruskal-Wallis tests^60^. Whenever the global statistical analysis including all treatments was statistically significant, individual post hoc comparisons were performed. An alpha value of 0.05 was considered as the statistical level of significance. The InfoStat v2012 statistical package was used for analyses (http://www.infostat.com.ar).

### Data availability

The datasets generated during and/or analyzed during the current study are available from the corresponding author on reasonable request.
